# Case of Hydrophobia

**Published:** 1854-07

**Authors:** T. D. Washburn


*Case of Hydrophobia.*—Reported by T. D. WASHBURN, M. D.

LAWRENCEVILLE, ILL., April 14th, 1854.

MESSRS. EDITORS: Allow me to present a case to your consideration, of peculiar interest to me. But to do justice to myself I shall be compelled to reverse the order of arrangement and come to the naked case at once and give you the history afterwards. I was extremely busy at the time and made no minute of the case, but its main features will not be easily obliterated. On the morning of the 13th of February last I was called some four miles in country to see a young lady who had been somewhat indisposed for two days previous. The most prominent symptom was continued pain in the head with general lassitude, until early this morning, awhile before day, she was taken with paroxysms of difficult breathing, the mother termed them "fainting spells" She had previously taken some purgative pills, which had operated more or less, but not given the slightest relief. The patient was of the nervous sanguine temperament, light hair, skin and eyes, in the 17th year of her age and had never menstrauted, though she had generally enjoyed good health.

On examination I found her breathing easily and quite natural, skin warm and moist; tongue moist and slightly furred; pulse soft; 95; bowels natural; no tenderness on pressure; in fact scarcely anything abnormal in her appearance or any functional derangement appreciable. On inquiry of her mother I learned that she had exhibited few if any premonitory symptoms of approaching catamenia, neither had she complained of any pain or suffering in her back during the present attack.

On returning to my patient to resume my observations, a slight rustling of the bed curtains threw her into a mild paroxysm of sobbing, catching her breath with considerable difficulty, expressing a good deal of anxiety in the countenance, at the same time springing from the resumbent to the semi-erect position to obtain relief, in three or four minutes it passed off and she was as natural as ever, her mother then informed me it was with great difficulty she could swallow, as it brought on one of these distressing paroxysms.

I acknowledge I was at some loss what course to persue in the premises. The young lady was perfectly sensible though possessing great nervous susceptibility, nether were there any physical signs of grave disease. Her tongue was somewhat coated, and the pain in the head might be dependent on some derangement of the bowels or torpor of the liver. (The farm was just bordering on an extensive marsh.) Her period of life and the absence of that function peculiar to her sex, seemed to indicate that some protean form of hysteria might develop itself and consequently the nervous system demand attention With theese views I prepared her Sub. Mur. Hydrag. and Jalap, of each ten grains, and administered it myself. Another paroxysm at once took place more severe than the former. With a wild but determined expression she opened her mouth, thrust her head forward to the spoon and closed her mouth convulsively on it. The larynx and muscles of deglutition seemed to be most involved, for she gasped her breath several times before she became easy. On attempting to take Water she became again convulsed, involving apparently the chest and throat, with a striking wild agony of expression, followed with profuse perspiration, but it lasted only a few minutes.

I then inquired of the mother if she had received any hurt or injury of any kind in the last few weeks and remarked that her symptoms resembled hydrophobia. She replied that she had not been hurt in any way as she knew and supposing she comprehended the term I used, I made no more definite inquiries in regard to it.

I then advised the use of sinapism to the spine and directed the following mixture to be given every four hours, viz: Ether, Tinct. Assafœtida, Camph., mixt ten grain of each. Dose, teaspoonful. Also to take Castor Oil if no action on the bowels by night.

Feb. 14th I visited her about the same hour, 9 o'clock, A. M., and found all the symptoms more aggravated, the paroxysms had continued to increase in frequency and violence. Though she had excessive thirst she dared not drink, neither had she taken the least nourishment and only a few doses of the medicine.

She had not closed her eyes all night and had been in a profuse sweat all the time during my absence, sitting up in bed most of the time.

The least breath of air or any attempt to administer anything or excitement or noise threw her into a spasm. Yet retaining her consciousness during the worst paroxysm. She still complains of pain in the head and constriction of the throat. She has had no action from the medicine. Her tongue remains about the same, moderately furred in the middle, but moist; pulse increased to 110, but not hard; extremities were rather of a clammy sweat and are cool; the kidneys perform their function; the expression rather more anxious, though the eye is natural; looks placid.

I attempted to apply some Chloroform and Turpentine to the region of the throat, but the first touch threw her into a violent spasm, with a wild agony of expression such as I have never witnessed, at the same time I noticed some slight convulsive motion about the face, but the agony of overwhelming fear (seeming to implore pity) depicted in the countenance, was unlike any convulsive disease I had ever seen.

There was so much about the case in its present appearance defying all ordinary medical treatment, at the same time portending evil, that I felt completely paralyzed and desired that council should be had as early in the evening as I could return. I then prepared her another active cathartic and directing her, to close her eyes and open her mouth, I placed the spoon between her lips when she seized it with a convulsive effort and swallowed the contents with great difficulty. It was the first she had taken since the evening previous. I then directed injections to be given after an interval of six hours and continued till the bowels were relieved, again remarking that there was much obscurity about the case and that the symptoms were strongly "Hydrophobic." (I suppose from the sequel that the term was not understood.)

Dr. McDonald, being designated as council, I called upon him upon my return to town and we made an arrangement to visit the case in the evening. But being unavoidably detained for some hours we were unable to see onr patient again before her decease, which took place at 6 P. M.

I learned from her mother afterwards that the paroxysms continued to increase and became more and more violent; that she would tremble or quiver all through the chest and call on those in the room to hold her about the short ribs and "bear as hard as they could." After 12 o'clock she became more restless and an hour or more before she expired commenced vomiting, which continued till the last Though suffering intense thirst she took nothing in her mouth till just a little while before she expired, when she drank a glass of ice water, telling them where they would find the ice and in fact perfectly conscious till the last.

Before the last paroxysm she called her connexions about the bed and bade them an affectionate farewell, telling them her next paroxysm would be the last and she preferred to die rather than live in such suffering. She was soon seized with one more violent than ever, and then became easy and sunk quietly into the sleep of death.

We come now to the unfortunate history preceding this event, though subsequently ascertained. On the day following her decease I learned from a connexion that a small pup had been brought into the family by this same young woman about the 1st of July last. In the course of a week or ten days it sickened and died as they supposed of distemper, though several times thinking it had something in its throat and was choaking, they attempted to remove it by putting their fingers in its mouth, all the members of the family were bitten during its illness, some slightly, and some enough to draw blood, though the mother informs me that she does not know whether she, the daughter, was bitten or not, and a single individual states that that this young lady said she was not bitten. But the dog was her's, and it is hardly probable she escaped when all the others were more or less wounded. On hearing the above circumstance, I immediately pronounced it a case of pure hydrophobia, yet I had never before witnessed one, and our standard authors are somewhat meagre on the subject.

On further investigation, I learned that the mother of the pup had probably been bitten several weeks (eight or ten) previous by a strange dog, which made great disturbance among the hogs and dogs during the night, but escaped before light, and visited the next neighbors and bit several hogs there. These hogs died, some fourteen or fifteen in number. The mother of this pup, some weeks after this occurrence, and when her pups (four in number) were some ten days old, manifested strange symptoms, was sullen, ran away from home, attacked a hog and bit it, which afterwards died, and was seen to bite her pups, and acted so strangely that the family thought it prudent to have her killed. It was about this time, just New Year, that this young lady, being on a visit to this place, took a fancy to this pup and took it home with her. Some one or two were bitten by the other pups, all which sickened and died in different localities, with similar symptoms, about the same time. With all these facts, the family in which the death took place do not believe it hydrophobia, and their confidence I judge is somewhat impaired in the attending physician, as since then another has been called in. Was the diagnosis correct? If not hydrophobia, what was it? Even though it could be proven that she was not bitten at all, I believe the least abraison or rupture of the cuticle would have been sufficient for her to become inoculated.

Its rapid and fatal termination points strongly towards its hydrophobic character. Its periodic paroxysms of laryngeal spasm,—the fearful agony of expression,—the continued consciousness,—the excitement which produced a paroxysm,—and the final vomiting,—all impress more deeply its hydrophobic character; and lastly, the canine origin can hardly be questioned. Even the girl's own aunt told her mother before she died that it must be hydrophobia; her symptoms reminded her so much of the manner in which their hogs died, which had been bitten by the mad dog. But I have trespassed already upon your time and patience. A single remark on treatment, and I will close, not entering into a further discussion of this subject at present.

No treatment in a well marked case of hydrophobia, so far as I can glean, ever seems to have been of much service, the most active, the most temporizing, and the most opposite, have alike been resorted to in vain. We should probably have attempted the use of chloroform in this case had we been able to have reached her earlier in the evening, though I believe the plan suggested by

Marshall Hall, of tracheotomy with an instrument designed specially for such cases, offers the best chances for success. (See Braithewaite, part 18, Art. 28.)

To those interested in unravelling some of the mazes of this formidable and terrific disease, I will refer them to Tweedie's Library of Practical Medicine, Good's Study of Medicine, and parts 13, 16, 18, 20, and 26 of Braithwaite's Retrospect.

NOTE.—The case so graphically described in the foregoing pages, is one clearly of Hydrophobia.

The probable affection of the animal, the irritation of the nervous system, by the sight or contact of fluids, the chronic character of the paroxysms, and above all the laryngeal spasm all indicate the specific character of the attack.—*Indiana Medical Journal.*

